# Preoperative Prediction Value of Pelvic Lymph Node Metastasis of Endometrial Cancer: Combining of ADC Value and Radiomics Features of the Primary Lesion and Clinical Parameters

**DOI:** 10.1155/2022/3335048

**Published:** 2022-06-30

**Authors:** Juan Bo, Haodong Jia, Yu Zhang, Baoyue Fu, Xueyan Jiang, Yulan Chen, Bin Shi, Xin Fang, Jiangning Dong

**Affiliations:** ^1^Department of Radiology, Anhui Provincial Hospital Affiliated to Anhui Medical University, Hefei, Anhui 230001, China; ^2^Department of Radiation Oncology, Anhui Provincial Hospital Affiliated to Anhui Medical University, Hefei, Anhui 230001, China; ^3^Bengbu Medical College, Bengbu, Anhui 233030, China; ^4^Department of Radiology, The First Affiliated Hospital of University of Science and Technology of China, Hefei, Anhui 230001, China

## Abstract

**Objective:**

To investigate the value of apparent diffusion coefficient (ADC) value of endometrial cancer (EC) primary lesion and magnetic resonance imaging (MRI) three-dimensional (3D) radiomics features combined with clinical parameters for preoperative prediction of pelvic lymph node metastasis (PLNM).

**Methods:**

A total of 136 patients with EC confirmed by postoperative pathology were retrospectively reviewed and analyzed. Patients were randomly divided into training set (*n* = 95) and test set (*n* = 41) at a ratio of 7 : 3. Radiomics features based on T_2_WI, DWI, and contrast-enhanced T_1_WI (CE-T_1_WI) sequence were extracted and screened, and then radiomics score (Rads-score) was calculated. Clinical parameters and ADC value of EC primary lesion were measured and collected, and their correlation with PLNM was analyzed. Receiver operating characteristic (ROC) curve was plotted to assess the diagnostic efficacy of the model. A nomogram for PLNM was created based on the multivariate logistic regression model.

**Results:**

The ADC value of the EC primary lesion showed inverse correlation with PLNM, while CA125 and Rads-score were positively associated with PLNM. A predictive model was proposed based on ADC value, Rads-score, CA125, and MR-reported pelvic lymph node status (PLNS) for PLNM in EC. The area under the curve (AUC) of the model is 0.940; the sensitivity and specificity (87.1% and 90.6%) of the model were significantly higher than that of the MRI morphological signs.

**Conclusion:**

A combination of ADC value, MRI 3D radiomics features of the EC primary lesion, and clinical parameters generated a prediction model for PLNM in EC and had a good diagnostic performance; it was a useful supplement to MR-reported PLNS based on MRI morphological signs.

## 1. Introduction

Endometrial cancer (EC) is one of the most common gynecological malignancies worldwide [1], and its morbidity and mortality are increasing over years. Lymph node metastasis (LNM) is one of the major metastatic routes of EC and the main adverse prognostic factor [2]. The overall five-year survival rate was significantly lower for those with LNM in EC compared to those without LNM [3]. Internationally, the use and the extent of lymphadenectomy in EC have been controversial. On the one hand, surgical complications pose significant challenges to EC patients. On the other hand, some studies have confirmed that low-risk patients do not benefit from lymphadenectomy. Emerging evidence confirmed the survival benefit of systematic lymphadenectomy in patients with EC with intermediate or high risk for LNM [4], suggesting that preoperative precise LN risk stratification is conducive to balancing the benefits of treatment and surgical complications. Therefore, preoperative prediction of LNM is crucial for EC treatment plan selection.

The occurrence of LNM implies the proliferation and spread of tumor, which is a complex process with multiple factors interacting with each other. Studies have reported that the intronic promoter p10 of TACC2 in primary lesion of EC is more active in those with LNM [5]. The transcriptional regulatory networks of EC primary lesion are different between LNM and non-LNM states [6], suggesting that LNM is closely related to the primary EC lesion. Therefore, preoperative evaluation of pelvic lymph node metastasis (PLNM) is theoretically feasible from the perspective of EC primary lesion. Conventional MRI mainly provides morphological information of LNs, such as LN size, enhancement mode, necrosis, signal characteristics, and extranodal expansion [7]. The morphologic appearance of metastatic LNs with short diameter is usually similar to that of nonmetastatic LNs, and the diagnostic process is subjective. Invasive sentinel node localization is not easy to implement due to the complexity of EC lymphatic drainage [8]. In addition, since about half of metastatic sentinels are small-volume metastases, most of them are not easily detected [9]. Therefore, it is necessary to find an accurate, effective, low-cost, and noninvasive method for preoperative assessment of PLNM in EC.

The apparent diffusion coefficient (ADC) value derived from diffusion-weighted imaging (DWI) can quantify the degree of restricted diffusion of water molecules. ADC value of EC differed among tumor grades and was significantly lower for patients with G2 and G3 than G1 [10]. A statistically significant negative correlation was observed between ADC value and the Ki-67 index [11]. Therefore, ADC value could be used as imaging biomarker to reflect the proliferative capacity and aggressiveness of tumors. Radiomics is a new data mining technique. It evaluates the inhomogeneity of image signals by quantifying the inhomogeneity and regularity of pixel gray values in normal and pathological tissues, through which it can reflect the microscopic heterogeneity at the histopathological level. Radiomics is potentially valuable for assessing tumor efficacy and predicting recurrence and metastasis [12–15]. Our study attempted to establish a predictive model for preoperative prediction of PLNM by combining the ADC value, MRI-based 3D radiomics features of EC primary lesion, and clinical parameters of patients.

## 2. Materials and Methods

### 2.1. Patients

**Figure 1:**
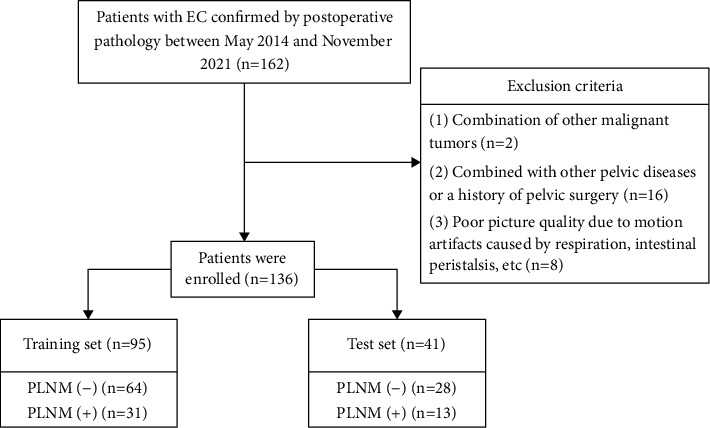
Flowchart of patient recruitment pathway.

This retrospective study with anonymous data was approved by the Ethics Committee of our hospital, and the informed consent requirement was waived. Data of 162 patients with EC confirmed by postoperative pathology from May 2014 to November 2021 were obtained by searching the picture archiving and communication system of our hospital.  Figure 1 showed the patient recruitment pathway. Inclusion criteria were (1) conventional MRI and DWI scan were performed one week before surgery, (2) radical hysterectomy, bilateral adnexectomy, and pelvic lymphadenectomy were performed in our hospital, (3) absence of any tumor-related treatment prior to imaging, and (4) maximum diameter of tumor (Mdot) ≥10.0 mm. Exclusion criteria were (1) combination of other malignant tumors, (2) combined with other pelvic diseases or a history of pelvic surgery, and (3) poor picture quality due to motion artifacts caused by respiration, intestinal peristalsis, and so on. Based on exclusion criteria, 136 patients were eventually included in our study. Patients were divided into training set (*n* = 95) and test set (*n* = 41) according to random distribution of 7 : 3. Based on the histopathologic examination, patients in training and test sets were further divided into PLNM (+) group and PLNM (−) group.

### 2.2. Imaging Protocol

Pelvic MRI was performed using a unit system (GE Signa HDXT 3.0T MRI scanner, GE Healthcare, USA) equipped with an eight-channel phased-array body coil. All patients fasted for at least 4 hours and were given an intramuscular injection of scopolamine hydrochloride half an hour before the MRI examination to reduce gastrointestinal peristalsis artifacts. Patients maintained a supine position with an empty urinary bladder. The scan covered the upper edge of the iliac crest to just below the pubic symphysis.

Detailed scanning parameters were listed in Table 1. The plain scan sequence includes axial fast spin-echo (FSE) T_1_-weighted images (T_1_WI), axial FSE T_2_-weighted images (T_2_WI), axial fat suppression (FS) FSE T_2_WI, and sagittal FSE T_2_WI. The enhancement sequence includes axial LAVA-FLEX in arterial phase, venous phase, and delayed phase, and sagittal LAVA-FLEX in late delayed phase. The delay time is 25 s, 60 s, 150 s, and 180 s, respectively. The *b* values of DWI are 0 and 1000 s/mm^2^. Contrast agent Gd-DTPA (Magnevist, Bayer Schering, Berlin, Germany) was injected through the anterior cubital vein with a high-pressure syringe at a flow rate of 2.5 ml/s at 0.1 mmol.

### 2.3. Surgical Procedure and Histopathology

Primary surgical treatment consisted of hysterectomy, bilateral adnexectomy, and pelvic lymphadenectomy. All surgical specimens were examined and reported by gynecologic pathologists. The 2014 World Health Organization (WHO) classification [16] and the 2018 revised FIGO staging criteria [17] for EC were used for histological diagnosis, grading, and pathological staging.

### 2.4. Imaging Analyses

**Figure 2:**
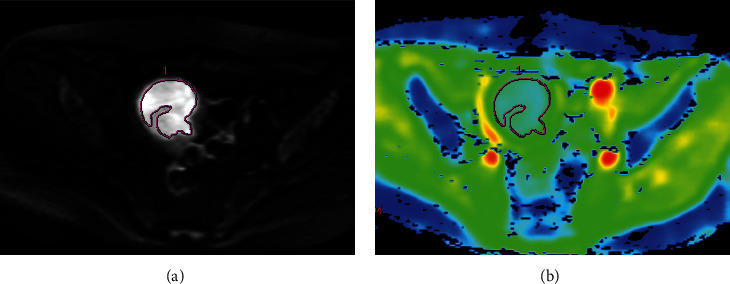
DWI image of a 57-year-old patient with EC of stage IB. (a) ROI was outlined at the largest tumor cross section on DWI (*b* = 1000 s/mm^2^). (b) ADC pseudocolor image showed that the ADC was 0.966 × 10^−3^ mm^2^/s.

**Figure 3:**
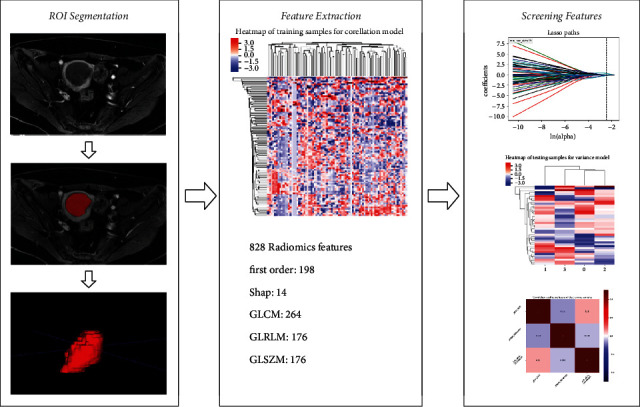
Radiomics workflow.

EC lesions and pelvic lymph node status (PLNS) were determined independently from MRI images by two attending physicians with 8 and 10 years of experience in gynecologic radiology, respectively. ADC value of the tumor were measured using GE Advantage Workstation 4.6, Function Tool software by the two attending physicians, respectively. The region of interest (ROI) was outlined at the largest tumor cross section and its two adjacent levels, and three ADC values were obtained and finally averaged, avoiding the areas of cystic change, hemorrhage, necrosis, and calcification as much as possible. The process of measuring the ADC value of EC primary lesion was shown in Figure 2. High-resolution axial T_2_WI, axial DWI (*b* = 1000 s/mm^2^), and axial contrast-enhanced T_1_-weighted (CE-T_1_WI) delayed-phase images of all patients were imported into ITK-SNAP (Version 3.6.0, http://www.itksnap.org) software in DICOM format, and the two attending physicians outlined the whole picture of the tumor layer by layer, and the ROIs should include degeneration, necrosis, and hemorrhage areas. The original images and ROIs were imported into AK software to extract radiomics features. 828 radiomics features were extracted from each sequence separately. The radiomics feature extraction and screening process was shown in Figure 3. The PLNS was evaluated based on MRI morphological signs, and PLNM was diagnosed if one of the following criteria was met: (1) short diameter of LNs in the axial plane ≥10.0 mm; (2) central necrosis or circumferential enhancement of LNs, (3) extra-peripheral invasion of LNs, including irregular enhancement of LN margins, blurring of surrounding fatty spaces, and fusion of LNs with each other. Two attending physicians performed the above steps twice separately, one week apart. Inter- and intragroup correlation coefficients and Cohen's kappa coefficient were calculated based on the two measurements for assessing consistency.

### 2.5. Statistical Analysis

Statistical analyses were implemented with R software (version 3.5.2; R Foundation for Statistical Computing, Vienna, Austria) and SPSS (version 24.0; IBM Corporation, Armonk, NY, USA). Agreement between two attending physicians in assessing PLN based on MRI images was assessed by calculating Cohen's kappa coefficient. The final consensus of two attending physicians was used for data analysis. The intraclass correlation coefficient (ICC) was used to evaluate the intra- and interobserver agreement of ADC value, radiomics parameters, and MR-reported Mdot. ADC value and MR-reported Mdot with higher intraobserver ICC were retained for subsequent data analysis. Radiometric features with intra- and interobserver ICC > 0.75 were retained for subsequent features screening.

Quantitative variables were tested for normality. Data were expressed as mean ± standard deviation (SD) when the distribution was normal and analyzed by independent samples *t*-test. Data conforming to nonnormal distribution were expressed as median and quartile and analyzed by Mann–Whitney *U* test. Categorical variables were expressed as composition ratios and analyzed by chi-square test or Fisher's exact test. A two-tailed *p* value <0.05 indicated statistical significance. The receiver operating characteristic (ROC) curves are plotted and the area under the curve (AUC) is calculated to assess the predictive power of the prediction model. DeLong's test was used to compare the AUCs between the prognostic models. The Hosmer–Lemeshow test was used to verify the goodness-of-fit of the prediction model. A nomogram for PLNM was created based on the multivariate logistic regression model. Adopting a calibration curve to measure the predictive performance of the model. Decision curve analysis was used to assess the clinical practicality of the model. Finally, the model performance was validated in the testing set.

## 3. Results

### 3.1. Intraobserver and Interobserver Agreement

The ranges of ICC (inter), ICC (intra), and Cohen's kappa coefficient were 0.791–0.939, 0.834–0.954, and 0.715, respectively, indicating that the intra- and interreproducibility is good.

### 3.2. General Clinicopathological Data, ADC Value, and Radiomics Features of Primary Lesion

The general clinicopathological data of the patients was shown in Table 2. A total of 136 patients with EC, aged 36–76 years, were included in this study. There were 44 patients in the PLNM (+) group and 92 patients in the PLNM (−) group. In the training and test sets, there was no statistical difference in age (*p*=0.196, 0.227, resp.) and menopausal status (*p*=0.675, 0.460, resp.) between the PLNM (+) and PLNM (−) groups. Adnexal metastasis, deep myometrial invasion, lymphatic vascular space infiltration (LVSI), and MR-reported PLNS were significantly more in the PLNM (+) group than in the PLNM (−) group. A significantly higher FIGO staging, CA125, and Ki-67 were found in PLNM (+) group, when compared to PLNM (−) group. MR-reported Mdot and pathological type were significantly different in the training set, while they were not significantly different in the test set (*p*=0.555, 0.443, resp.).

The radiomics features extraction process and results are as follows: (1) 828 radiomics features were extracted from each image sequence (T_2_WI, DWI, and CE-T_1_WI) of each patient, and a total of 2484 features were extracted from each patient. (2) 1890 features with good stability (ICC > 0.75) were retained. (3) A *z*-score standardization was applied to each feature. (4) The least absolute shrinkage and selection operator (LASSO) algorithm was used for filtering and 10 radiomics parameters were screened with |*γ*| < 0.7. (5) Four radiomics features were obtained by multivariate logistic regression as independent discriminant features. (6) Rads-score was calculated based on linear combinations of regression coefficients. Compared to the PLNM (−) group, the PLNM (+) group had significantly lower ADC value and higher Rads-score. The ADC value, radiomics features, and Rads-score for the training and test sets were shown in Table 3.

### 3.3. Diagnostic Efficacy of the Predictive Model

Age at onset was divided into ≥54 years and <54 years groups and the MR-reported Mdot was divided into ≥4 cm and <4 cm groups according to the median. The ADC value and Rads-score were divided into two groups according to the cut-off values. We investigated the association of CA125, ADC value, MR-reported Mdot, MR-reported PLNS, menopausal status, age at onset, and Rads-score with PLNM using univariate analysis. CA125, ADC value, MR-reported PLNS, MR-reported Mdot, and Rads-score were significantly associated with PLNM. A multivariate analysis of these factors showed that CA125(OR = 6.971; 95% CI = 1.545–31.443; *p*=0.012), ADC value (OR = 0.004; 95% CI = 0.000–0.427; *p*=0.020), and Rads-score (OR = 1.721; 95% CI = 1.139–2.601; *p*=0.010) were independent risk factors for PLNM. The higher the ADC value, the higher the likelihood of not developing PLNM in EC patients. The higher the CA125 and Rads-score, the higher the risk of PLNM (Table 4). In addition, the MRI-reported PLNS (OR = 9.126; 95% CI = 2.043–40.759; *p*=0.004) also reflected the risk of PLNM to some extent.

**Figure 4:**
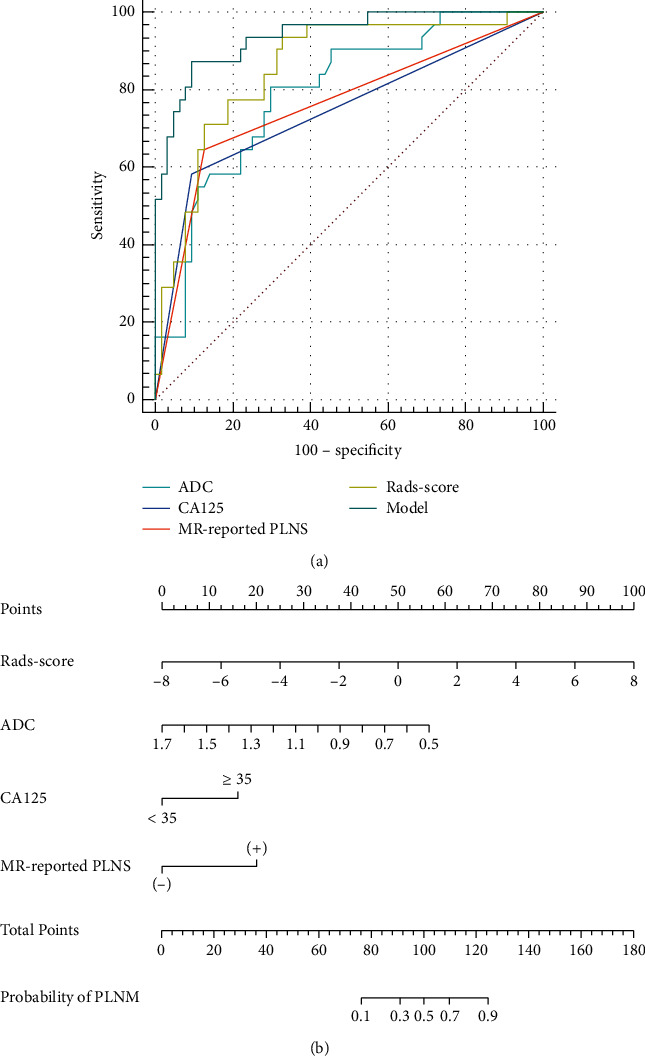
(a) ROC curves of the ADC, CA125, MR-reported PLNS, Rads-score, and model in the training set. AUCs of ADC, CA125, MR-reported PLNS, Rads-score, and model were 0.791, 0.743, 0.760, 0.855, and 0.940 in the training set, respectively. (b) Nomogram of the model for PLNM in EC in the training set.

**Figure 5:**
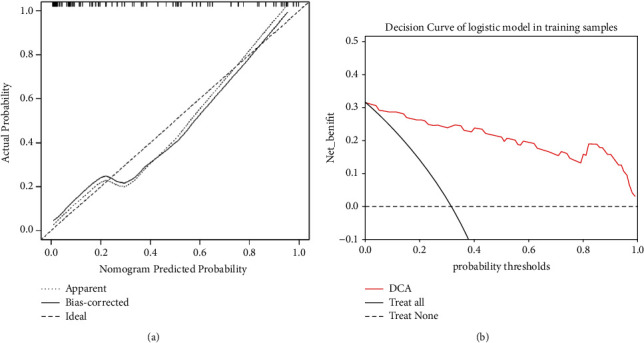
(a) Calibration curves for nomogram in the training set. The *x*-axis is the predicted probability of the nomogram; the *y*-axis is the actual probability of occurrence of PLNM. Perfect prediction corresponds to the “Ideal” line, the “Apparent” line represents the entire cohort (*n* = 95), and the “Bias-corrected” line represents the performance of the nomogram obtained by bootstrapping (*B* = 1000 repetitions). (b) The decision curve of nomogram of the model in the training set.

The following cut-off values were obtained by ROC curve analysis and used to distinguish PLNM (+) from PLNM (−): ADC = 0.908 × 10^−3^ mm^2^/s, Rads-score = −1.161. The AUC of the Rads-score was 0.855, the highest among all predictive parameters. In the training set, the AUC, sensitivity, and specificity of the prediction model established by combining ADC value, clinical parameters, and radiomics features (0.940, 87.1%, and 90.6% resp.) were all higher than MRI morphological signs (0.760, 64.5%, and 87.5%, resp.) (Table 5 and Figure 4(a)). DeLong's test showed that the diagnostic efficacy of the prediction model was better than that of the other single parameter in both the training and test sets (Table 6). The nomogram of the prediction model based on CA125, ADC value of EC primary lesion, MR-reported PLNS, and Rads-score in the training group was shown in Figure 4(b). The nonsignificant statistic of the Hosmer–Lemeshow test (*p*=0.815) indicated that the training set prediction model was well fitted; calibration curves for nomogram in the training set were shown in Figure 5(a). The decision curve analysis showed that model could add a net benefit to the treat-all or treat-none scheme (Figure 5(b)).

## 4. Discussion

Independent risk factors for EC lymph node metastasis include positive peritoneal cytology, deep myometrial invasion, LVSI, and FIGO staging [18, 19]. However, this data was usually available from the postoperative pathology, so the conditions for preoperative evaluation of PLNM were limited. In this study, we developed a predictive model based on accessible and available clinical parameters (CA125 and MR-reported PLNS), ADC value of the primary lesion, and Rads-score. It showed a good diagnostic efficacy (AUC = 0.940) and the sensitivity (87.1%) and specificity (90.6%) were greatly improved compared with the MRI morphological signs. Furthermore, we developed and validated a nomogram for this prediction model to facilitate individualized and noninvasive preoperative assessment of the PLNM in EC.

Conventional MRI remained the main tool for preoperative assessment of LNM in EC, but daily diagnosis remained at the macromorphology stage. ADC value was negatively correlated with the cell density of tumors. Investigators found ADC value of LNs was unable to differentiate benign LNs from malignant LNs [20, 21]. However, several reports showed that the ADC value of breast lesions in patients with positive axillary LN metastasis was significantly lower than those with negative axillary LNM [22]. Therefore, our study focused on the ADC value of EC primary lesion in different LN states and found that ADC value showed inverse correlation with PLNM. The optimal diagnostic threshold for ADC value was (0.908 × 10^–3^ mm^2^/s) in our study. Its sensitivity, specificity, and accuracy were 80.7%, 72.6%, and 71.6%, respectively. In fact, the reason for the lower ADC value of EC primary lesions in PLNM (+) group is not clear. However, in our study, the Ki-67 indexes of PLNM (+) group were higher than those of PLNM (−) group. Ki-67 is a marker of cell proliferation, suggesting that EC tumor cells in the PLNM (+) group had higher proliferative activity, stronger invasiveness, and higher cell density. It may be the reason for the lower ADC value in the PLNM (+) group.

Metastasis is closely related to the heterogeneity of malignant tumors, and radiomics features can quantify the microscopic heterogeneity at the histopathological level [23, 24]. MRI-based radiomics has been considered a reliable tool for accurate preoperative assessment of LNM in several cancers, such as cervical cancer [25], rectal cancer [15], and oral tongue squamous carcinoma [26]. From this, we developed a Rads-score based on 3D radiomics features from axial T_2_WI, axial DWI (*b* = 1000 s/mm^2^), and axial CE-T1WI delay period to improve diagnostic efficiency. In our study, 4 features with predictive value for PLNM were screened from 2484 radiomics features (glcm_MCC@CE-T1WI, shape_MajorAxisLength@T_2_WI, HLL_firstorder_Kurtosis@DWI, and glcm_Correlation@DWI). The glcm_MCC reflected the complexity of textures. The shape_MajorAxisLength described the difference in the geometric shape of the two groups of lesions, and it was independent of the gray level intensity distribution in the ROI. HLL_firstorder_Kurtosis was a measure of the “peakness” of the distribution of values in the image ROI. The glcm_Correlation showed the linear dependency of gray level values to their respective voxels in the GLCM. Multifactorial logistic regression analysis was performed on the 4 obtained characteristics, and Rads-score was calculated based on linear combinations of regression coefficients. In our study, the AUC, sensitivity, and specificity of Rads-score in predicting PLNM were 0.855, 93.6%, and 67.2%, respectively, suggesting that radiomics had promising clinical applications in the preoperative evaluation of PLNM in EC.

It is undeniable that the most intuitive way to assess PLNM was based on LN morphology. However, the present study found a high specificity of 87.5% but low sensitivity of 64.5% based solely on morphological characteristics of LN. This may be due to the difficulty in distinguishing benign enlarged LN (e.g., infection, granulomatous disease, and reactive hyperplasia) from metastatic LN based on morphology alone [27]. The independent diagnostic efficacy of MRI-reported PLNS in this study was low (AUC = 0.760). In clinical practice, CA125 has been routinely used to diagnose a variety of malignancies, especially epithelial ovarian cancer [28]. Quan et al. [29] demonstrated that serum CA125 significantly correlated with clinicopathological risk factors, such as depth of myometrial invasion, LN status, and FIGO stage and that CA125 is a promising prognostic indicator for EC [30, 31]. In our study, CA125 could be used as an independent predictor of PLNM in EC patients, which is consistent with the results of Lei et al. [32]. However, its sensitivity is not high, which may be related to the vulnerability of CA125 to other factors, such as menstruation, pregnancy, endometriosis, and peritonitis [33].

Significantly, we found that age at onset, menopause, and MR-reported Mdot were not risk factors for PLNM. The age of patients and the MR-reported Mdot in the PLNM (+) group were slightly greater than those in the PLNM (−) group, but the difference was not statistically significant. EC usually occurs after menopause [34]. The patients in this study were relatively concentrated in age, mainly between 50 and 60 years. Most of these patients have reached the age of menopause; menopause was not associated with PLNM also.

In this study, the nomogram model combining ADC value, MRI-based 3D radiomics features of the EC primary lesion, and clinical parameters had a good predictive performance for PLNM in EC. Probably, because ADC value indirectly reflected the proliferative status of cells in the EC primary lesion, clinical parameters such as CA125 reflected the biological behavior of EC to some extent, and radiomics features reflected the microscopic characteristics and heterogeneity of EC, so the nomogram model could predict PLNM in EC from different perspectives and had a better performance.

The limitations of this study include three aspects. First, in this retrospective study, the model was only validated with an internal independent test group, and external validation and prospective validation were not performed. Second, the relationship between the microcirculatory status of EC primary foci and PLNM was not analyzed in this study, while the microangiogenic status of EC primary lesion may be related to PLNM, which will be further explored in the future by combining intravoxel incoherent motion diffusion-weighted imaging (IVIM-DWI) and dynamic contrast-enhanced MRI (DCE-MRI).

## 5. Conclusion

In conclusion, the predictive model based on ADC value, MRI 3D radiomics features of primary lesion, and clinical parameters showed promising performance for preoperative evaluation of PLNM in EC. It had higher diagnostic efficiency compared to MR-reported PLNS based on MRI morphological signs and contributed to clinical decision-making and improving prognosis further.

## Figures and Tables

**Table 1:** MRI scanning protocols.

Sequence	TR/TE	FOV	Matrix	Slice gap (mm)	Slice thickness (mm)
Axial T_1_WI	500/7.2	38 cm × 26 cm	352 × 192	2	6
Axial T_2_WI	4600/68	24 cm × 24 cm	320 × 256	2	6
Axial (FS)-T_2_WI	4600/68	24 cm × 24 cm	320 × 256	2	6
Sagittal T_2_WI	4600/68	26 cm × 24 cm	320 × 256	2	6
Axial T_1_WI LAVA-FLEX	36/1.3	38 cm × 36 cm	320 × 224	0	4
Sagittal T_1_WI LAVA-FLEX	36/1.3	28 cm × 26 cm	320 × 224	0	4
Axial DWI	4000/65	38 cm × 26 cm	96 × 130	1	4

**Table 2:** Selected clinicopathological data of patients with endometrial cancer.

Characteristics	Training set		*p*	Test set		*p*	*p*
	PLNM (+)	PLNM (−)		PLNM (+)	PLNM (−)		
Age at onset (years)	55 (51, 63)	53 (49, 61.8)	0.196	54 (49.5, 59)	53 (49, 57.5)	0.227	0.132

CA125 (U/mol)			<0.001			0.007	<0.001
≥35	18 (58.1%)	6 (9.4%)		76.9 (%)	9 (32.1%)		
<35	13 (41.9%)	58 (90.6%)		3 (23.1%)	19 (67.9%)		

MR-reported Mdot (cm)	5 (3.5, 5.5)	4 (3, 5)	0.034	5 (2.9, 6)	4.3 (2.9, 5.8)	0.555	0.034

MR-reported PLNS			0.009			0.069	<0.001
MR-reported PLNM (+)	20 (64.5%)	8 (12.5%)		6 (46.2%)	4 (14.3%)		
MR-reported PLNM (−)	11 (35.5%)	56 (87.5%)		7 (53.8%)	24 (85.7%)		

LVSI			<0.001			0.002	<0.001
Present	17 (54.8%)	4 (6.20%)		8 (61.5%)	3 (10.7%)		
Absent	14 (45.2%)	60 (93.8%)		5 (38.5%)	25 (89.3%)		

Ki-67(%)	60 (50, 70)	50 (30, 70)	0.014	70 (60, 75)	45 (32.5, 67.5)	0.005	<0.001

Adnexal metastasis			<0.001			0.024	<0.001
Present	13 (41.9%)	5 (7.80%)		5 (38.5%)	2 (7.10%)		
Absent	18 (58.1%)	59 (92.2%)		8 (61.5%)	26 (92.9%)		

Dmi			<0.001			<0.001	<0.001
Present	21 (67.7%)	14 (21.9%)		10 (76.9%)	8 (28.6%)		
Absent	10 (32.3%)	50 (78.1%)		3 (23.1%)	20 (71.4%)		

Menopause			0.675			0.460	0.912
Present	17 (54.8%)	38 (59.4%)		9 (69.2%)	16 (57.1%)		
Absent	14 (45.2%)	26 (40.6%)		4 (30.8%)	12 (42.9%)		

FIGO stage			<0.001			<0.001	<0.001
I–II	0	56 (87.5%)		0	25 (89.3%)		
IIIA	0	8 (12.5%)		0	3 (10.7%)		
IIIC	25 (80.6%)	0		9 (69.2%)	0		
IVA	6 (19.4%)	0		2 (15.4%)	0		
IVB	0	0		2 (15.4%)	0		

Pathological types			0.036			0.443	0.017
Endometrioid	19 (61.3%)	52 (81.2%)		8 (61.5%)	22 (78.6%)		
Nonendometrioid	12 (38.7%)	12 (18.8%)		5 (38.5%)	6 (21.4%)		

*Note.* CA125 = carcinoma antigen 125; MR-reported Mdot = MR-reported maximum diameter of tumor; MR-reported; PLNS = MR-reported pelvic lymph node status; LVSI = lymphatic vascular space infiltration; and Dmi = deep myometrial invasion.

**Table 3:** The ADC value, radiomics features, and Rads-score of the training and test sets.

Characteristics	Training set		*p*	Test set		*p*	*p*
	PLNM (+)	PLNM (−)		PLNM (+)	PLNM (−)		
ADC (×10^−3^ mm^2^/s)	0.810 (0.780, 0.901)	1.001 (0.878, 1.205)	<0.001	0.884 (0.783, 0.906)	0.930 (0.889, 1.099)	0.006	<0.001
glcm_MCC @ CE-T_1_WI	0.736 ± 0.110	0.661 ± 0.102	0.001	0.767 ± 0.095	0.684 ± 0.087	0.008	<0.001
shape_MajorAxisLength@T_2_WI	49.972 (38.648, 62.845)	37.884 (31.643, 46.827)	0.001	57.149 (45.914, 103.431)	39.518 (34.102, 48.604)	0.002	<0.001
HLL_firstorder_Kurtosis@DWI	3.563 (2.946, 4.140)	4.056 (3.388, 4.805)	0.011	3.991 (3.387, 4.727)	5.122 (4.534, 5.671)	0.002	0.009
glcm_Correlation@DWI	0.334 (0.190, 0.387)	0.284 (0.118, 0.346)	0.002	0.297 (0.275, 0.399)	0.263 (0.143, 0.323)	0.019	0.008
Rads-score	0.741 ± 2.323	−2.290 ± 2.190	<0.001	0.0746 ± 2.612	−1.751 ± 2.308	0.029	<0.001

**Table 4:** Univariate and multivariate logistic regression analyses for ADC value of EC primary lesion, clinical parameters, and Rads-score in estimating PLNM.

Parameters	Univariate analysis			Multivariate analysis		
	OR	95% CI	*p*	OR	95% CI	*p*
CA125 (≥35 U/mol/<35 U/mol)	13.385	4.444–40.308	<0.001	6.971	1.545–31.443	0.012
Age at onset (≥54/<54)	1.037	0.982–1.095	0.192			
Menopause (present/absent)	0.831	0.350–1.974	0.675			
MR-reported Mdot (≥4 cm/<4 cm)	1.324	1.022–1.715	0.033			
ADC (≥0.908 × 10^−3^ mm^2^/s/<0.908 × 10^−3^ mm^2^/s)	0.001	0.000–0.030	<0.001	0.004	0.000–0.427	0.020
MR-reported PLNS (present/absent)	12.727	4.480–35.155	<0.001	9.126	2.043–40.759	0.004
Rads-score (≥−1.161/<−1.161)	2.215	1.536–3.195	<0.001	1.721	1.139–2.601	0.010

**Table 5:** Diagnostic performance of single parameters and predictive model for predicting PLNM in EC.

Parameters	Training set				Test set			
	AUC	Sensitivity (%)	Specificity (%)	Accuracy (%)	AUC	Sensitivity (%)	Specificity (%)	Accuracy (%)
CA125	0.743	58.1	90.6	80.0	0.724	76.9	67.9	70.7
ADC	0.791	80.7	72.6	71.6	0.772	84.6	64.3	68.3
MR-reported PLNS	0.760	64.5	87.5	80.0	0.659	46.2	85.7	73.1
Rads-score	0.855	93.6	67.2	74.7	0.728	61.54	82.1	73.2
Model	0.940	87.1	90.6	88.4	0.918	92.3	89.3	85.4

**Table 6:** AUCs of the four parameters and the predictive model were compared.

	Training set	Test set
ADC *vs.*CA125	0.474	0.686
ADC *vs.* MR-reported PLNS	0.625	0.358
ADC *vs.* Rads-score	0.286	0.678
ADC *vs.* Model	<0.001	0.080
CA125 *vs.* MR-reported PLNS	0.816	0.525
CA125 *vs.* Rads-score	0.047	0.973
CA125 *vs.* Model	<0.001	0.007
MR-reported PLNS *vs.* Rads-score	0.138	0.637
MR-reported PLNS *vs.* model	<0.001	0.002
Rads-score *vs.* model	0.005	0.025

## Data Availability

The data used to support the findings of this study are available from the corresponding author upon request.
